# Single‐cell sequencing of multi‐region resolves geospatial architecture and therapeutic target of endothelial cells in esophageal squamous cell carcinoma

**DOI:** 10.1002/ctm2.1487

**Published:** 2023-11-21

**Authors:** Jiacheng Dai, Xiaoxiang Xi, Zidong Liu, Weicheng Wu, Sibo Zhu, Xiaoyang Zhang, Yuwei Huang, Jiayue Meng, Liyun Yuan, Chen Suo, Jiangli Xue, Ziyu Yuan, Ming Lv, Weimin Ye, Li Jin, Guoqing Zhang, Xingdong Chen

**Affiliations:** ^1^ State Key Laboratory of Genetic Engineering, Human Phenome Institute, Zhangjiang Fudan International Innovation Center, School of Life Science Fudan University Shanghai China; ^2^ Taixing People's Hospital Taizhou China; ^3^ Fudan University Taizhou Institute of Health Sciences Taizhou China; ^4^ Bio‐Med Big Data Center, Key Laboratory of Computational Biology, Shanghai Institute of Nutrition and Health University of Chinese Academy of Science, Chinese Academy of Science Shanghai China; ^5^ Department of Epidemiology and Ministry of Education Key Laboratory of Public Health Safety, School of Public Health Fudan University Shanghai China; ^6^ Clinical Epidemiology Unit Qilu Hospital of Shandong University Jinan China; ^7^ Department of Epidemiology and Health Statistics, School of Public Health, Key Laboratory of Ministry of Education for Gastrointestinal Cancer Fujian Medical University Fuzhou China; ^8^ Yiwu Research Institute of Fudan University Yiwu China; ^9^ Department of Oncological Science Huntsman Cancer Institute, University of Utah, Salt Lake City Utah USA; ^10^ National Clinical Research Center for Aging and Medicine Huashan Hospital Fudan University Shanghai China

Dear Editor,

Esophageal squamous cell carcinoma (ESCC) is the predominant subtype of esophageal cancer in Asia, which is characterized by rapid invasion and metastasis and has a poor clinical outcome.^1^ Recently, the genetic landscape^2^ and single‐cell atlas^3^, ^4^ of ESCC have been unveiled; however, the vascular heterogeneity in ESCC remained unknown. Our study fully characterized the endothelial cells in ESCC, which may have implications for the development of anti‐angiogenic therapies (AATs) in ESCC.

To interrogate the cellular heterogeneity and evolutionary trajectory, we performed scRNA‐seq on cells from three geospatial regions, including 20 tumours and 31 matched normal esophageal tissues with defined distances from the tumour edge (i.e., adjacent, 2 cm from tumour edge; distant, 5 cm from tumour edge; Figure 1A). After quality control, a total of 206 380 cells were included for subsequent analysis (Figure S1A). Cells clustered into 10 major cell lineages (Figure 1B), highlighting the gene signatures of major cell lineages (Figure 1C). These cells were adequately derived from all geospatial regions, and the compositions of lineages varied across the ESCC and peri‐tumoural tissue (Figure 1D). Hierarchical clustering revealed that the expression patterns of epithelial cells, fibroblasts, and endothelial cells from the tumours were different from those of cells from their matched geospatial normal counterpart (Figure 1E; Figure S1B). Analysis of geospatial cell composition identified topological gradients of the cell lineages from the distant tissue to tumours (Figure 1F–G).

**FIGURE 1:**
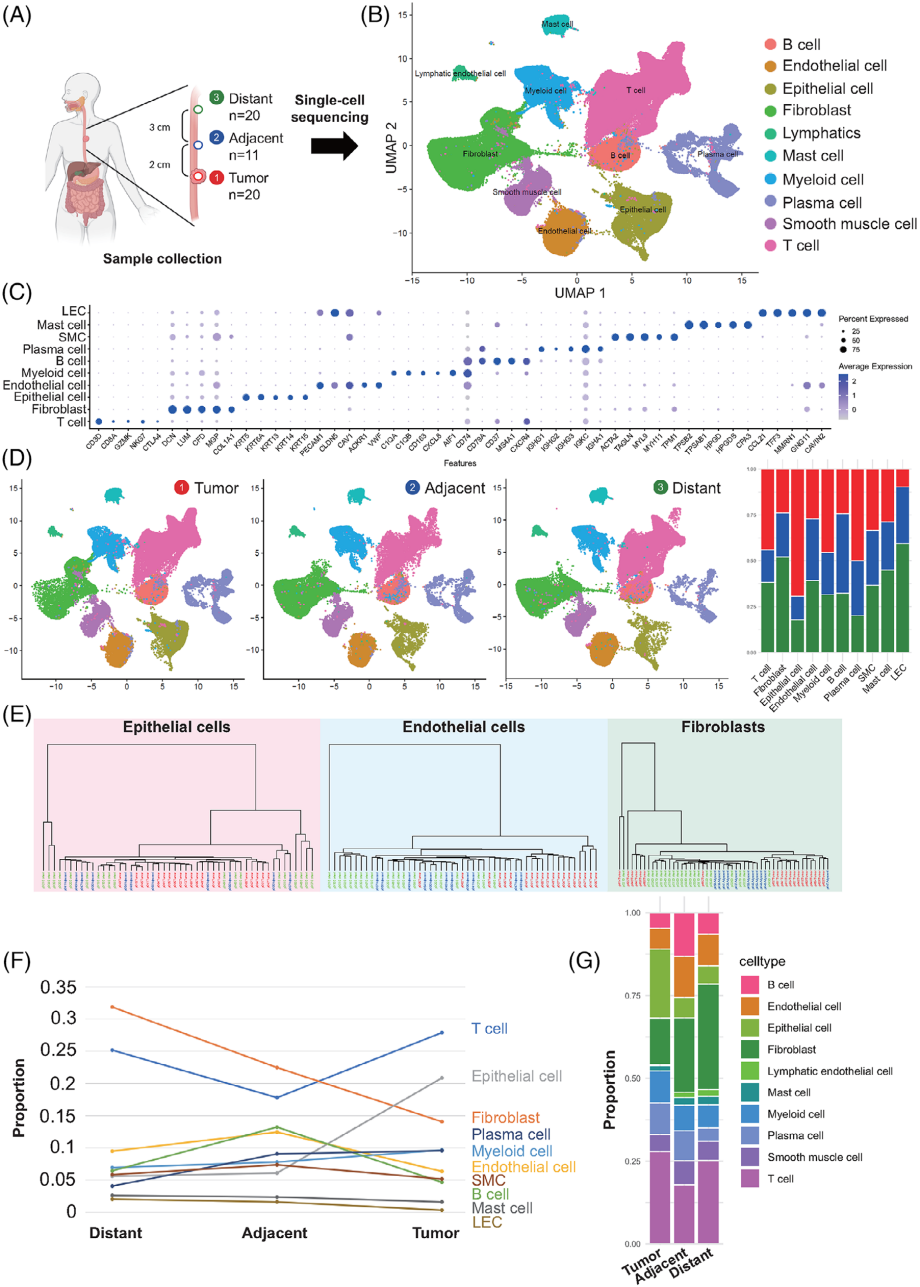
Single‐cell atlas of esophageal squamous cell carcinoma (ESCC)and the peritumoural tissue. (A) Schematic diagram showing the sampling strategy of ESCC participants for analysis of scRNA‐seq. (B) UMAP plot of included cells from all ESCC patients. Cells are colored according to assumed cell types. (C) Bubble plot showing the expression levels of marker genes in each cell type. (D) Plot of cell types and fractions (stacked bar plots) by different geospatial locations in UMAP. Colors represent cell types as in (B). (E) Dendrograms showing hierarchical relationships of expression patterns of epithelial cells, endothelial cells and fibroblasts from each patient's geospatial locations. (F) Line plot showing the changes in the relative proportions of each cell types among geospatial locations. (G) Bar plot showing the relative percentages of each cell types among the geospatial regions.

To further investigate the taxonomy of ECs in ESCC, we re‐clustered vascular ECs and identified 16 EC subtypes (Figure 2A). We annotated the clusters based on the expression of canonical gene signatures of artery, capillary, vein, and tumour endothelial cells (TECs) (Figure 2B). The cell composition of EC subtypes varied across different regions, but the angiogenic TECs were highly enriched in ESCC (Figure 2C), for example, COL4A1^+^ tip cells (*p* < .05; Figure 2D). We found that tip cells in ESCC account for over 25% of ECs in tumours, compared to previously reported tip cells compromised only a minority (<10%) of ECs in lung tumour.^5^ However, the correlation between TEC composition and cancer stage was insignificant in our study (Figure S2A), which deserved further investigation. We further identified COL4A1^+^ tip cells, RGCC^+^ stalk cells, and UNC5B^+^ arteries with highly expressed genes associated with collagen modification, angiogenesis, and vascular endothelial growth factor (VEGF) signaling (Figure 2E; Figure S2B, C) in line with previous observations.^5^, ^6^ Notably, these angiogenic TECs were mostly derived from tumour tissues (Figure 2E) and highly expressed VEGFR1 and VEGFR2 (Figure 2F), which were promising targets for anti‐VEGF therapy.^7^ Besides, by co‐localizing COL4A1 and CD31 using immunostaining, it was validated that tip cells were present in the tumours rather than in adjacent samples (Figure S2D). Last, the validation of a high fraction of TECs and the interpretation of other EC subtypes were present in supplementary.

**FIGURE 2:**
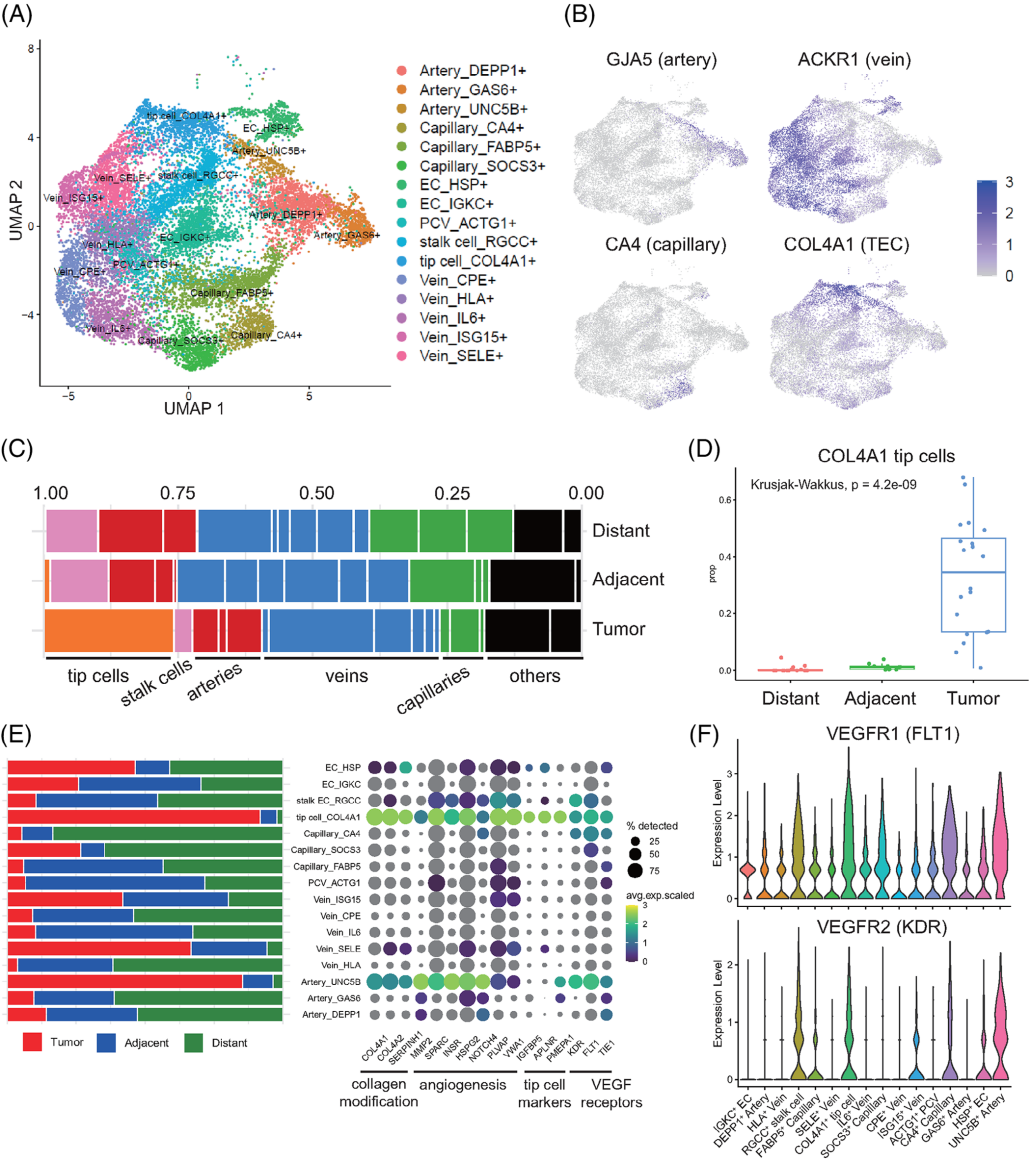
Overview of endothelial cell atlas in esophageal squamous cell carcinoma (ESCC). (A) UMAP visualization of ECs colored by their assigned cell types. (B) UMAP plot color‐coded for the expression of canonical marker genes for vascular ECs. (C) Stacked bar plot showing the cell proportions of EC subtypes by geospatial regions. The relative division scaled to 100%. (D) Box plot showing the cell proportion of tip cells by geospatial regions. Statistical method is Wilcox‐test, *p* > .05. (E) Bobble plot showing the expression level and the expressed cell proportions of selected gene signatures for angiogenic ECs. Stacked bar plot in the left showing the relative contribution of sampling regions to the cell proportions. The relative contribution scaled to 100%. (F) Violin plot showing the expression levels of the indicated genes in angiogenic ECs.

Multi‐regional or geospatial analyses have been employed to interrogate cell evolution,^8^ so we investigate the differentiation trajectory of tip cells in ESCC. The pseudo‐time analysis showed that RGCC^+^ stalk cells, UNC5B^+^ arteries, and SELE^+^ veins are the sources of the COL4A1^+^ tip cells (Figure 3A), demonstrating that tip cells originate from different vascular beds. Focusing on the formation of tip cells, the dynamic model inferred a cascade of gene expression events underlying the process (Figure 3B). For example, genes associated with collagen modification (COL4A1 and COL4A2) and angiogenesis (PDGFB) were highly expressed in the early stage, while tumour‐associated marker PMEPA1 was expressed in the late stage. Next, we investigated the underlying activated transcription factors that drive the maturity of tip cells. We highlighted that *ETS1* regulons were activated in angiogenic TECs (Figure 3C), as previously reported *ETS1* is essential for angiogenesis.^7^, ^9^ Besides, the expression of these regulons was significantly associated with the gene of dynamic events of tip cells (e.g., VEGFR1, COL4A1, and FAM167B), suggesting the regulons activate the gene expression of tip cells in the early stage (Figure 3D). Last, we found the high expression of the dynamic event's genes, FAM167B and DYSF, had shorter overall survival in ESCC patients (Figure 3E). In summary, these results identified dynamic trajectories and distinct transcription factors that highlighted *ETS1* may drive the differentiation processes from different EC subtypes to tip cells, and provided a rich resource to encourage further functional validation.

**FIGURE 3:**
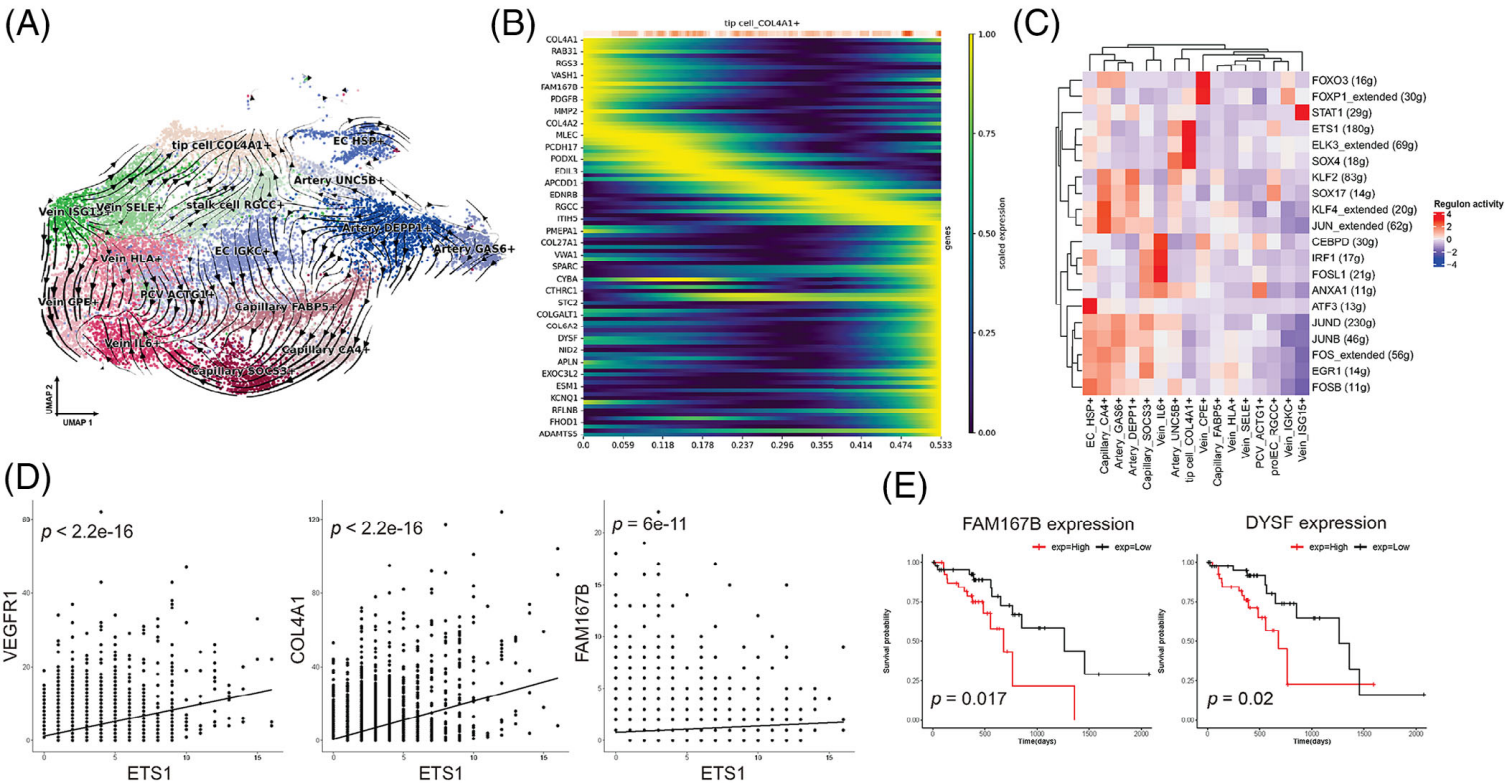
Differentiation trajectory and key regulons of endothelial cells. (A) UMAP of ECs with scVelo‐projected velocities, shown as streamlines. Arrows represent the direction of cells’ flow. (B) Heatmap showing the gene expression trends of the top genes which have significant correlation of the expression level with the differentiation probabilities of COL4A1^+^ tip cell. (C) Heatmap of key regulons of all EC subtypes in esophageal squamous cell carcinoma (ESCC). (D) Scatter plot showing correlation of the expression level of *ETS1* and VEGFR1 (FLT1), COL4A1, and FAM167B. Statistical method is *Pearson* correlation, *p* < .05. (E) Overall survival of 82 ESCC patients selected from the TCGA dataset and stratified by the indicated genes of tip cells.

Finally, we integrated single‐cell data of ECs from ESCC and non‐small cell lung cancer (NSCLC)^5^ to compare the EC phenotypes (Figure 4A). The visual plot showed that cells were well mixed rather than grouped by dataset‐ or tissue‐specific conditions (Figure 4B). We observed that tip cells of lung and esophagus represent substantial transcriptome similarity. The hierarchical clustering showed that the tip cells of ESCC and NSCLC were more likely to cluster together (Figure 4C). Also, the marker genes of EC subsets between ESCC and NSCLC were compared, showing a significant overlap of marker genes between angiogenic EC clusters in lung‐ and esophageal cancer (Figure 4D). Besides, we calculated the potential regulon of TECs in lung, revealing regulon *ETS1* was also upregulated in tip cells in lung (Figure 4E). Furthermore, the upregulation of regulons *KLF2* and *ELK3* were associated with shorter overall survival in LUSC patients (Figure 4F). In summary, these results revealed a comparable transcriptome overlap of angiogenic ECs in ESCC and NSCLC.

**FIGURE 4:**
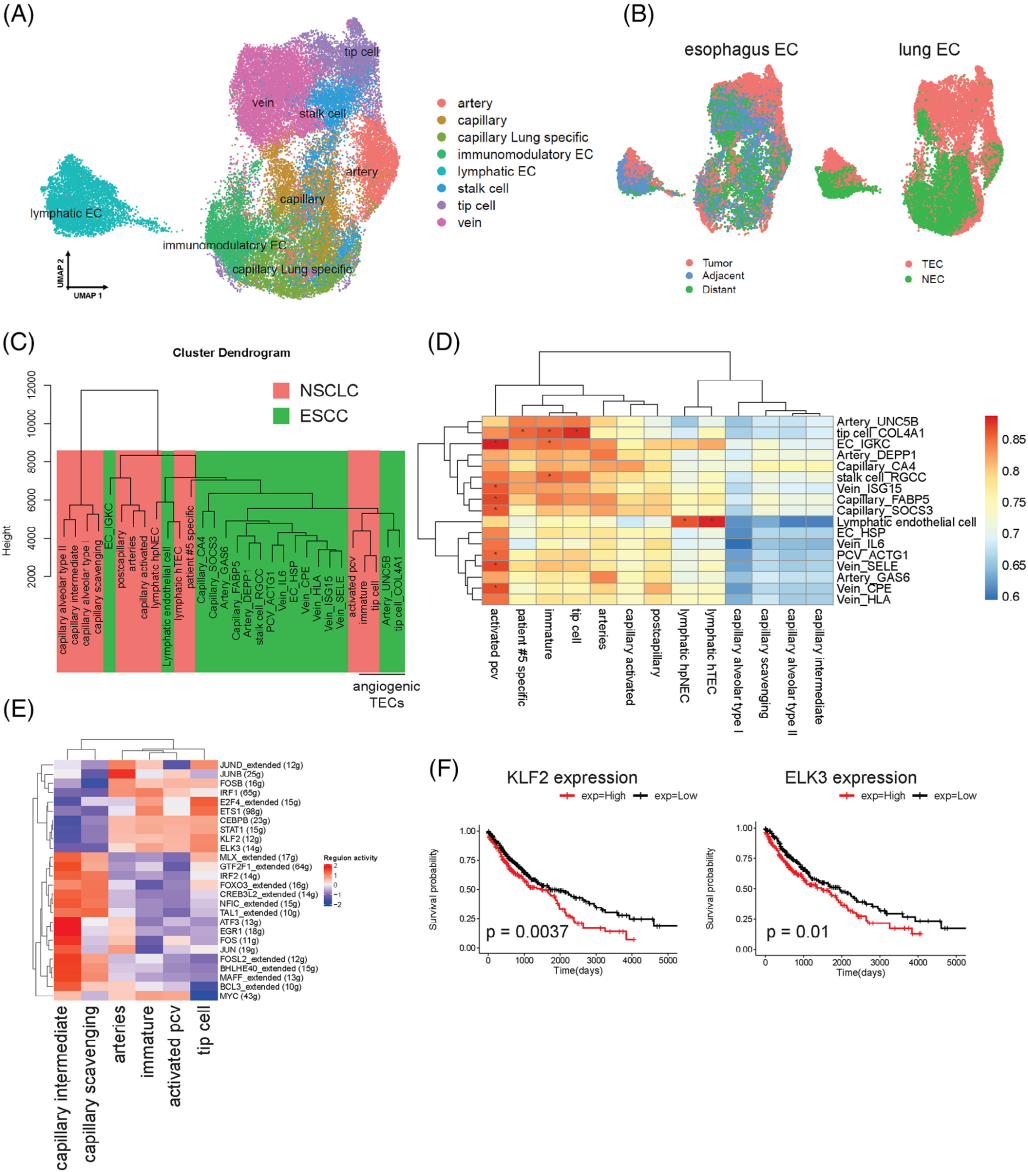
Meta‐analysis of endothelial cells across tumour type. (A) UMAP plot of all ECs integrated from the lung and esophagus datasets. Cells are colored by cell types inferred by meta‐analysis. (B) UMAP visualization of cells colored geospatial locations divided by datasets. (C) Dendrograms showing hierarchical relationships of the identities of endothelial cells from the lung and esophagus datasets. (D) Heatmap of EC phenotype correlation by gene signatures showing a transcriptomic congruency in esophageal squamous cell carcinoma (ESCC) and NSCLC. Shown data are Spearman r‐value, *adjust *p* < .05 (FDR). (E) Heatmap of key regulons of EC subtypes in NSCLC. (F) Overall survival of 542 LUSC patients selected from the TCGA dataset and stratified by the key regulons of tip cells in (E).

In conclusion, our results provided a geospatial framework for characterizing the vascular architecture in ESCC, highlighting the diverse EC identities, differentiation trajectories, and high resolution of cell heterogeneity. Our analysis identified *ETS1* as key regulons that regulate the expression events in tip cell differentiation, which provided a rich resource to encourage further functional validations on associations between TFs and angiogenesis. Our integrated analysis of lung and esophagus identified substantial transcriptome similarity of tip cells across tumour type, suggesting the potential clinical implications of AATs in ESCC.^10^

## Supporting information

Supporting Information

(**A**) Quality control of single‐cell data by filtering aberrantly high gene count and extensive mitochondrial contamination. Violin plot showing the number of unique genes (nFeature_RNA), the total number of molecules (nCount_RNA), per cent of mitochondrial RNA and ribosome RNA of datasets before (left) and after (right) the quality control.
(**B**) Dendrograms showing hierarchical relationships of cells among the geospatial locations based on the computed Euclidean distance using transcriptomic features. Dendrograms are shown for other cell types by patients.

(**A**) Box plot showing the cell proportion of tip cells by cancer stages of ESCC patients. Statistical method is Wilcox‐test, *p* > .05.
(**B** and **C**) Different colored bar plots showing the functional pathways enriched by gene signatures of tip cells and stalk cells.
(**D**) Representative micrograph of COL4A1^+^ tip cells for ESCC tumour (left) and corresponding adjacent tissue (right) section immunostained for CD31 and COL4A1. Nuclei were stained with DAPI. Scale bar, 50 μm.

(**A**) Stacked bar plot showing the relative fraction of EC subtypes in tumour region of digestive system tumour. Different color indicated different EC subtype.
(**B**) Stacked bar plot showing cell proportions of detailed EC subtypes in another ESCC dataset (GSE160269).
(**C**) UMAP visualization showing EC subtypes colored by assigned cell types.
(**D**) Bubble plot showing the expression level and the expressed cell proportions of selected gene signatures.

(**A**) Bubble plot showing the expression level and the expressed cell proportions of selected gene signatures in arteries.
(**B**) Colored bar plots showing the functional pathways enriched by gene signatures of UNC5B^+^ artery.
(**C**) Bubble plot showing the expression level and the expressed cell proportions of selected gene signatures in capillaries.
(**D** and **E**) Colored bar plots showing the functional pathways enriched by gene signatures of CA4^+^ capillary and SOCS3^+^ capillary.
(**F**) Bubble plot showing the expression level and the expressed cell proportions of selected gene signatures in veins.
(**G** to **L**) Colored bar plots showing the functional pathways enriched by gene signatures of HLA^+^ vein (G), IL6^+^ vein (H), CPE^+^ vein (I), ISG15^+^ vein (J), ACTG1^+^ post‐capillary vein (K), and SELE^+^ vein (L).

(**A**) Box plot showing the cell proportion of tumour‐associated ECs increased by geospatial regions. Statistical method is Wilcox‐test, *p* < .05.
(**B**) Box plot showing the cell proportion of immunomodulatory veins decreased by geospatial regions. Statistical method is Wilcox‐test, *p* < .05.

Supporting Information

Supporting Information
