# Supplementary material for: Single‐cell sequencing of multi‐region resolves geospatial architecture and therapeutic target of endothelial cells in esophageal squamous cell carcinoma
Source: Clin Transl Med. 2023 Nov 21;13(11):e1487. doi: 10.1002/ctm2.1487 (PMC10660795; doi:10.1002/ctm2.1487)
Supplement: Supplementary file 1 — Supporting Information [file CTM2-13-e1487-s001.docx]

**Supplementary Appendix**

**Results**

**High Cell Fraction of Tip Cells in Digestive System Tumor**

As we found a higher fraction of tip cells (~25%) in tumors of esophageal squamous cell carcinoma (ESCC) than that in tumors of non-small cell lung carcinoma (NSCLC) (<10%), we wondered whether the cell fraction of tip cells varied in different cancer type. Therefore, we evaluated the cell fraction of tumor endothelial cells (TECs) in digestive system tumor, including the colon, pancreas, liver (Hepatocellular carcinoma (HCC) and intrahepatic cholangiocarcinoma (ICC)), and esophagus (squamous cell carcinoma) (Method). We extracted the endothelial cell (EC) subpopulations (PECAM1-positive) from all cells and re-clustered the ECs. We found that tip cells of digestive system tumors account for over 25% of total ECs in tumors (Figure S3A). We also validated that tip cells in ESCC account for over 25% of total ECs in tumors using another ESCC single-cell dataset^1^ (Figure S3B). To validate our discoveries, we further clustered the EC populations of ESCC in a higher resolution (Figure S3C). Notably, consistent EC identity and gene signatures were identified, such as tip cell, UNC5B^+^ artery, and immunomodulatory vein (Figure S3D), suggesting the robustness of our discoveries in ESCC. In summary, these results found a higher fraction of tip cells in digestive system tumors.

**Single-Cell Atlas of EC Phenotypes in Human Esophagus Cancer**

Given the design of multi-regional sampling, we identified different EC subpopulations that presumed normal. Arterial ECs highly expressed canonical gene signatures SOX17, GJA5, and JAG1. These arterial EC subtypes were characterized by high expression of DEPP1, GAS6, or UNC5B (Figure S4A). Notably, the high expressed genes in UNC5B^+^ artery involved in blood vessel branching (DLL4, NOTCH4, COL4A1, CTNNB1, ENG), suggesting a potential role of arteries in angiogenesis (Figure S4B). Capillary ECs highly express CA4 as canonical gene signatures (Figure S4C). The highly express genes of capillary ECs involved in lipid transport (FABP4, FABP5), lipopolysaccharide response, and fatty acid metabolic process (Figure S4D). The SOCS3^+^ capillary was involved explicitly in the JAK-STAT signaling pathway (Figure S4E), suggesting potential functions in inflammation. Distinct venous EC subtypes were identified, which shared upregulation of canonical gene signatures ACKR1 and VWF and characterized by different gene expressions (Figure S4F). For instance, HLA^+^ vein involved in MHC-II-mediated antigen presentation (Figure S4G); IL6^+^ vein involved in interleukin response (Figure S4H); CPE+ vein involved in leukocyte and neutrophil migration (Figure S4I); ISG15^+^ vein involved in interferon response (Figure S4J). The functions of these venous ECs were like the scavenging ECs and immunomodulatory ECs that reported previously^2,3^. In addition, postcapillary vein (ACTG1^+^ PCV highly expressed ACKR1 and CAV1) and SELE^+^ vein upregulated gene signatures involved in hypoxia response and wound healing (Figure S4K-L), suggesting that vascular ECs in peri-tumoral locations were activated for wound healing.

We further revealed the changes of cell proportion of the EC subtypes. We noted tumor-associated endothelial cells (tip cell and UNC5B artery) and SELE^+^ veins were mostly derived from tumors (>50%); the cell proportions of these cells were significantly higher with increasing geospatial proximity to tumors (Figure S5A). In contrast, the cell proportions of immunomodulatory veins were significantly lower with increasing geospatial proximity to tumors (Figure S5B).

**Materials and methods**

**Human subjects.** Study patients were evaluated at Taizhou People’s Hospital and underwent standard-of-care surgical resection of ESCC. All samples in the study were obtained under the approval of the ethics review boards of School of Life Sciences of Fudan University (No. FE21011). All applicable institutional regulations concerning the ethical use of information and samples from human volunteers were confirmed to be strictly followed in this study. Each patient provided written informed consent. Details on the gender, age, and clinical data of the patients included are shown in Table S1.

**Single-cell library construction and sequencing.** Following the surgical resection, the esophagus tissues from the tumor, adjacent, and distant regions were taken and immediately transported on ice. Upon arriving research facility, samples were minced and transferred to enzymatic digestion (Liberase™, Roche, Cat#5401119001). Cell suspension and pellets were further dispersed by pipetting and filtered via a 40μm Mesh (Falcon, Cat#352340). Then, cells were sorted by FACS (BD FACSAria III). The single-cell suspensions were converted to separate barcoded scRNA-seq libraries using the library (Chromium GEM single cell 3’ Kit, 10x Genomics). For each patient, all samples were processed in parallel with the same protocol. Next, libraries were sequenced using Novaseq 6000 platforms. The gene expression matrices with the CellRanger software (version 3.0.2) using the 10X human transcriptome GRCh38-3.00 as the reference genome. Data from the raw unfiltered matrix was further processed using R (version 3.6.0).

**Single-cell RNA-seq data analysis.** The suggested integration workflow of Seurat (version 3.2.0) was used in this study. The following quality control steps were used: cells expressing <200 genes (low quality), >5000 genes (potential doublets), or >10% of unique molecular identifiers derived from the mitochondrial genome were removed. Data were normalized and scaled with the “SCTransform” function, and the expression of mitochondrial or ribosome gene were normalized with the parameter “vars.to.regress”. Then, Principal component analysis (PCA) was performed using the “RunPCA” function with the number of PCs set to 100. A shared nearest neighbor (SNN) graph was constructed using the “FindNeighbors” function with the 100 PCs, then cells were clustered by the “FindClusters” function. The resolution parameter (Res) set to 1 for most clustering analysis if not particularly indicated. Specifically, for meta-analysis of ECs of lung and esophagus, the Res set to 2. The “RunUMAP” function was used for the visualization plot with the “umap-learn” method, setting “dims” to 1:100. Marker genes for each cluster were detected using the “FindAllMarkers” function, setting the parameter ‘min.pct’ to 0.25. The cell clusters were manually annotated according to these marker genes. Finally, the similar cell clusters were merged and visualized on a UMAP plot. To study the transcriptome pattern among cell types, the pairwise Spearman correlations were calculated from the average expression levels, which were calculated using the “AverageExpression” function in Seurat; then the Euclidean distances were calculated, and hierarchical clustering were used with method “average”. Last, the figures were visualized using “as.dendrogram” and “plot” function.

**Single-cell validation data of digestive system tumor.** The single-cell data of endothelial cells of digestive system tumor were obtained from PreDigs (https://www.biosino.org/predigs/). We included three colon adenocarcinoma (GSE132465^4^, GSE144735^4^, and E-MTAB-8107^5^), one esophageal cancer (GSE160269^1^), two hepatocellular carcinomas (GSE125449^6^ and GSE112271^7^), one intrahepatic cholangiocarcinoma (GSE138709^8^), and one pancreas cancer (CRA001166^9^). The resolution parameter (Res) set to 0.5 for the individual analysis of digestive system tumor.

**Doublet simulation and cell pruning.** Potential doublets of all cells were simulated with DoubletFinder^10^ (version 2.0.3) using the Seurat object. The doublet inference was calculated for each donor separately to avoid the influence of batch or sequence pipeline. The parameters of “pK_value” and “nExp_poi” were calculated for each patient. The putative singlets of cells were kept and used for downstream analyses.

**Cell-type annotation.** Cell clusters were annotated manually to the major cell types according to known markers and marker genes for each cluster. A brief description of cell-type annotation for each lineage is provided below. **Endothelial lineage cells** (PECAM1, CDH5)^11,12^ were substantially divided into arterial, venous, capillaries, lymphatic endothelium, as well as endothelial cells with proliferative potentials, termed tumor endothelial cells (tip-like and stalk-like cells). Canonical marker genes of these EC subpopulations were shown in Table S4.

Table S4. list of endothelial cell canonical marker gene

| Gene | Cell lineage | Species | Source | Reference |
| --- | --- | --- | --- | --- |
| SOX17 | Artery | Human and mouse | Immunostaining experiment | ^13-15^ |
| GJA5 | Artery | Human and mouse | Knockout experiment | ^16,17^ |
| EFNB2 | Artery | Human and mouse | Knockout experiment | ^18,19^ |
| ACKR1 | Vein | Human and mouse | Immunostaining experiment | ^20,21^ |
| CA4 | Capillary | Human and mouse | Immunohistochemistry | ^22^ |
| PROX1 | Lymphatic | Human and mouse | Knock in experiment | ^23,24^ |
| LYVE1 | Lymphatic | Human and mouse | Immunohistochemistry | ^25^ |
| COL4A1 | Tip-like | Human and mouse | scRNA-seq | ^26,27^ |
| HSPG2 | Tip-like | Human and mouse | scRNA-seq | ^26,27^ |
| PMEPA1 | Tip-like | Human and mouse | scRNA-seq | ^26,27^ |
| SELP | Stalk-like | Human and mouse | scRNA-seq | ^26^ |
| AQP1 | Stalk-like | Human and mouse | scRNA-seq | ^26^ |

**Fibroblasts** were generally selected by marker genes, including COL1A1, COL3A1, COL5A1, and COL6A1^28^. **T lineage cells** were determined as the cluster of cells with expression of T cell receptor component CD3D, CD4 and CD8A^29^. **B lineage cells** were determined as the cluster of cells with expression of CD79A and MS4A1. Most B/plasma cells highly expressed Immunoglobulin related genes (IGKC, IGLC2, etc.)^30^. **Epithelial lineage cells** were identified by marker genes included KRT5, KRT13, KRT14, and KRT17. **Myeloid lineage cells** were identified by marker genes, including macrophage (CD163, CD86, C1QA, C1QB) and neutrophil (CD15, CD16, and S100A8)^31^. **Mast cells** were identified by marker genes TPSB2 and TPSAB1^32^. **Smooth muscle cells** were identified by marker genes, including TAGLN^33^, ACTA2^34^, and MYH11^35^. Pericyte were identified by reported markers^35^.

**Statistical analysis.** All statistical analyses were performed using R (version 3.6.0). Log-rank tests and Kaplan-Meier plots were used for the survival analysis. Gene Ontology enrichment for biological processes, molecular functions, and cellular components were performed using the ClusterProfiler package^36^ (version 3.18.0). All statistical results were considered significant at P<0.05. The Benjamini-Hochberg (BH) method was applied to control the false discovery rate (FDR) and to calculate adjusted P values (q-values).

**Immunohistochemistry.** Formalin-fixed paraffin-embedded human esophagus tissue slides (5 μm) were subjected to immunofluorescence. The slides were deparaffinized, rehydrated, and permeabilized using 0.1% Triton-X 100 for 10 min, then boiled at 95-98 °C for 10 min in Tris-EDTA antigen retrieval solution. Slides were further blocked with 5% normal donkey serum for 2 h at room temperature. Slides were incubated with the primary antibody for CD31(1:200; #14-0311-82, eBioscience, San Diego, CA) and COL4A1 (1:50; #SAB4300825, Sigma-Aldrich, Darmstadt, Germany) diluted in Antibody Diluent (#36323ES60, Yeasen, China) overnight at 4 °C. The next day, slides were incubated with secondary antibody conjugated with Alexa Fluor (Yeasen). Slides were digitalized on an Olympus FV3000 confocal microscopy (Tokyo, Japan).

**Differentiation trajectory inference.** To reveal the differentiation trajectory, the RNA-velocity tool, scVelo^37^ and CellRank^38^ were used. The splicing information was extracted from the bam files generated by CellRanger. The resulting loom files were merged and transformed into h5ad files. The information of UMAP, cluster, and cell type was extracted from Seurat object correspondingly. The output of the scVelo script was visualized by function “velocity_embedding_stream”. The terminal and initial states were automatically identified by the CellRank using the function “terminal_states” and the parameter “n_states = 4”. Initial and terminal states were visualized on a two-dimensional embedding.

**TF-target interaction inference.** The TF regulatory network was inferred by SCENIC^39^ (version 1.2.4) package. The RcisTarget database used hg38 reference for the motif score (Motif collection version 9: 24k motifs). Key TFs were shown by the function “regulonAUC” and “regulonActivity_byCellType”, then the key TFs were used to plot the heatmap.

**Data integration and meta-cluster identification.** The scRNA-seq dataset of endothelial cells from tumoral human lung tissue was used for validation. Raw counts and the corresponding metadata were downloaded from https://www.vibcancer.be/software-tools/lungTumor_ECTax. To integrate EC datasets from ESCC and NSCLC, the pipeline of batch correction was used to remove the technical differences. First, the raw count data matrix in each dataset was scaled to z-score. Then, the z-score transformed gene expression matrices were combined by column and only genes present in two datasets were kept. Next, Harmony^40^ was applied after PCA, and the UMAP and clustering (both implemented in the Seurat v3 pipeline) were performed based on the harmony feature instead of PCA feature. Subsequently, the Seurat v3 pipeline was performed. The resolution parameter for the combined datasets was set to 0.5. The similar cell clusters were merged. The heatmap was displayed based on the average expression level of the original clusters of each study and used spearman correlation. The most significant correlation results were highlighted with star.

**Data sharing statement.** The data that support the findings of this study are available at NODE (http://www.biosino.org/node/project/detail/OEP003180) or GSA-human (https://ngdc.cncb.ac.cn/gsa-human/) from the corresponding author upon reasonable request. Code used for the analysis of scRNA-seq data is available at GitHub (https://github.com/Scathacheng/ECatlas).

**Funding.** This work was supported by the National Natural Science Foundation of China (grant numbers: 82122060, 82073637, 91846302), the National Key Research and Development Program of China (grant number: 2019YFC1315804, 2017YFC0907000, 2021YFC2501800), the Innovation Grant from Science and Technology Commission of Shanghai Municipality, China (grant number: 20ZR1405600), three-Year Action Plan for Strengthening Public Health System in Shanghai (grant number: GWV-10.2-YQ32), Shanghai Municipal Science and Technology Major Project (grant number: 2017SHZDZX01), and Grant of Science and Technology of Fujian, China (grant number: 2019L3006).

**Reference**

1 Zhang, X. *et al.* Dissecting esophageal squamous-cell carcinoma ecosystem by single-cell transcriptomic analysis. *Nat Commun* **12**, 5291, doi:10.1038/s41467-021-25539-x (2021).

2 Goveia, J. *et al.* An integrated gene expression landscape profiling approach to identify lung tumor endothelial cell heterogeneity and angiogenic candidates. *Cancer Cell* **37**, 21-36. e13 (2020).

3 Geldhof, V. *et al.* Single cell atlas identifies lipid-processing and immunomodulatory endothelial cells in healthy and malignant breast. *Nature communications* **13**, 1-19 (2022).

4 Lee, H.-O. *et al.* Lineage-dependent gene expression programs influence the immune landscape of colorectal cancer. *Nature genetics* **52**, 594-603 (2020).

5 Qian, J. *et al.* A pan-cancer blueprint of the heterogeneous tumor microenvironment revealed by single-cell profiling. *Cell research* **30**, 745-762 (2020).

6 Ma, L. *et al.* Tumor cell biodiversity drives microenvironmental reprogramming in liver cancer. *Cancer cell* **36**, 418-430. e416 (2019).

7 Losic, B. *et al.* Intratumoral heterogeneity and clonal evolution in liver cancer. *Nature communications* **11**, 291 (2020).

8 Zhang, M. *et al.* Single-cell transcriptomic architecture and intercellular crosstalk of human intrahepatic cholangiocarcinoma. *Journal of hepatology* **73**, 1118-1130 (2020).

9 Peng, J. *et al.* Single-cell RNA-seq highlights intra-tumoral heterogeneity and malignant progression in pancreatic ductal adenocarcinoma. *Cell research* **29**, 725-738 (2019).

10 McGinnis, C. S., Murrow, L. M. & Gartner, Z. J. DoubletFinder: doublet detection in single-cell RNA sequencing data using artificial nearest neighbors. *Cell systems* **8**, 329-337. e324 (2019).

11 Newman, P. J. The biology of PECAM-1. *J Clin Invest* **100**, S25-29 (1997).

12 Dejana, E. & Vestweber, D. The role of VE-cadherin in vascular morphogenesis and permeability control. *Prog Mol Biol Transl Sci* **116**, 119-144, doi:10.1016/b978-0-12-394311-8.00006-6 (2013).

13 Trimm, E. & Red-Horse, K. Vascular endothelial cell development and diversity. *Nature Reviews Cardiology* **20**, 197-210 (2023).

14 Corada, M. *et al.* Sox17 is indispensable for acquisition and maintenance of arterial identity. *Nature communications* **4**, 1-14 (2013).

15 González-Hernández, S. *et al.* Sox17 controls emergence and remodeling of nestin-expressing coronary vessels. *Circulation research* **127**, e252-e270 (2020).

16 Lu, X. J. & Wang, H. T. Reduced Gja5 expression in arterial endothelial cells impairs arteriogenesis during acute ischemic cardiovascular disease. *Experimental and Therapeutic Medicine* **14**, 4339-4343 (2017).

17 Phansalkar, R. *et al.* Coronary blood vessels from distinct origins converge to equivalent states during mouse and human development. *Elife* **10**, e70246 (2021).

18 Wang, H. U., Chen, Z.-F. & Anderson, D. J. Molecular distinction and angiogenic interaction between embryonic arteries and veins revealed by ephrin-B2 and its receptor Eph-B4. *Cell* **93**, 741-753 (1998).

19 Korff, T., Braun, J., Pfaff, D., Augustin, H. G. & Hecker, M. Role of ephrinB2 expression in endothelial cells during arteriogenesis: impact on smooth muscle cell migration and monocyte recruitment. *Blood, The Journal of the American Society of Hematology* **112**, 73-81 (2008).

20 Thiriot, A. *et al.* Differential DARC/ACKR1 expression distinguishes venular from non-venular endothelial cells in murine tissues. *BMC biology* **15**, 1-19 (2017).

21 Guo, X. *et al.* Endothelial ACKR1 is induced by neutrophil contact and down-regulated by secretion in extracellular vesicles. *Frontiers in Immunology* **14**, 1181016 (2023).

22 Fleming, R. E., Crouch, E. C., Ruzicka, C. A. & Sly, W. S. Pulmonary carbonic anhydrase IV: developmental regulation and cell-specific expression in the capillary endothelium. *American Journal of Physiology-Lung Cellular and Molecular Physiology* **265**, L627-L635 (1993).

23 Hong, Y. K. *et al.* Prox1 is a master control gene in the program specifying lymphatic endothelial cell fate. *Developmental dynamics: an official publication of the American Association of Anatomists* **225**, 351-357 (2002).

24 Wilting, J. *et al.* The transcription factor Prox1 is a marker for lymphatic endothelial cells in normal and diseased human tissues. *The FASEB Journal* **16**, 1271-1273 (2002).

25 Jackson, D. G., Prevo, R., Clasper, S. & Banerji, S. LYVE-1, the lymphatic system and tumor lymphangiogenesis. *Trends in immunology* **22**, 317-321 (2001).

26 Zhao, Q. *et al.* Single-cell transcriptome analyses reveal endothelial cell heterogeneity in tumors and changes following antiangiogenic treatment. *Cancer research* **78**, 2370-2382 (2018).

27 Zeng, Q. *et al.* Understanding tumour endothelial cell heterogeneity and function from single-cell omics. *Nature Reviews Cancer*, 1-21 (2023).

28 Muhl, L. *et al.* Single-cell analysis uncovers fibroblast heterogeneity and criteria for fibroblast and mural cell identification and discrimination. *Nature communications* **11**, 1-18 (2020).

29 Zheng, L. *et al.* Pan-cancer single-cell landscape of tumor-infiltrating T cells. *Science* **374**, abe6474 (2021).

30 Sanz, I. *et al.* Challenges and opportunities for consistent classification of human B cell and plasma cell populations. *Frontiers in immunology* **10**, 2458 (2019).

31 Cheng, S. *et al.* A pan-cancer single-cell transcriptional atlas of tumor infiltrating myeloid cells. *Cell* **184**, 792-809. e723 (2021).

32 Maaninka, K., Lappalainen, J. & Kovanen, P. T. Human mast cells arise from a common circulating progenitor. *Journal of allergy and clinical immunology* **132**, 463-469. e463 (2013).

33 Milewicz, D. M. *et al.* Altered smooth muscle cell force generation as a driver of thoracic aortic aneurysms and dissections. *Arteriosclerosis, thrombosis, and vascular biology* **37**, 26-34 (2017).

34 Alexander, M. R. & Owens, G. K. Epigenetic control of smooth muscle cell differentiation and phenotypic switching in vascular development and disease. *Annual review of physiology* **74**, 13-40 (2012).

35 Baek, S.-H. *et al.* Single cell transcriptomic analysis reveals organ specific pericyte markers and identities. *Frontiers in Cardiovascular Medicine* **9** (2022).

36 Yu, G., Wang, L.-G., Han, Y. & He, Q.-Y. clusterProfiler: an R package for comparing biological themes among gene clusters. *Omics: a journal of integrative biology* **16**, 284-287 (2012).

37 Bergen, V., Lange, M., Peidli, S., Wolf, F. A. & Theis, F. J. Generalizing RNA velocity to transient cell states through dynamical modeling. *Nature biotechnology* **38**, 1408-1414 (2020).

38 Lange, M. *et al.* CellRank for directed single-cell fate mapping. *Nature methods* **19**, 159-170 (2022).

39 Aibar, S. *et al.* SCENIC: single-cell regulatory network inference and clustering. *Nature methods* **14**, 1083-1086 (2017).

40 Korsunsky, I. *et al.* Fast, sensitive and accurate integration of single-cell data with Harmony. *Nature methods* **16**, 1289-1296 (2019).
